# High synthetic cost-amino acids reduce member interactions of acetate-degrading methanogenic microbial community

**DOI:** 10.3389/fmicb.2024.1368215

**Published:** 2024-03-28

**Authors:** Jian Yao, Quan Zhang, Min Gou, Yue-Qin Tang

**Affiliations:** ^1^College of Architecture and Environment, Sichuan University, Chengdu, Sichuan, China; ^2^Sichuan Environmental Protection Key Laboratory of Organic Wastes Valorization, Chengdu, Sichuan, China; ^3^SINOPEC (Dalian) Research Institute of Petroleum and Petrochemicals Co., Ltd., Dalian, Liaoning, China

**Keywords:** amino acid availability, acetate-degrading methanogenic microbial community, amino acid synthesis cost, microbiological interactions, microbial community structure

## Abstract

**Introduction:**

The cooperation among members of microbial communities based on the exchange of public goods such as 20 protein amino acids (AAs) has attracted widespread attention. However, little is known about how AAs availability affects interactions among members of complex microbial communities and the structure and function of a community.

**Methods:**

To investigate this question, trace amounts of AAs combinations with different synthetic costs (low-cost, medium-cost, high-cost, and all 20 AAs) were supplemented separately to acetate-degrading thermophilic methanogenic reactors, and the differences in microbial community structure and co-occurring networks of main members were compared to a control reactor without AA supplementation.

**Results:**

The structure of the microbial community and the interaction of community members were influenced by AAs supplementation and the AAs with different synthetic costs had different impacts. The number of nodes, links, positive links, and the average degree of nodes in the co-occurrence network of the microbial communities with AAs supplementation was significantly lower than that of the control without AAs supplementation, especially for all 20 AAs supplementation followed by the medium- and high-cost AAs supplementation. The average proportion of positive interactions of microbial members in the systems supplemented with low-cost, medium-cost, high-cost, all AAs, and the control group were 0.42, 0.38, 0.15, 0.4, and 0.45, respectively. In addition, the ecological functions of community members possibly changed with the supplementation of different cost AAs.

**Discussion:**

These findings highlight the effects of AAs availability on the interactions among members of complex microbial communities, as well as on community function.

## 1 Introduction

Microorganisms in nature do not exist alone but often share a habitat with many other microorganisms. These microorganisms have very complex interactions with each other, which are critical for the stability of microbial community structure and ecological functions (Zengler and Zaramela, 2018). Currently, researchers have been trying to understand the interactions among individual microorganisms through mathematical modeling and construction of artificial laboratory floras, and one of the major research hotspots is the study of amino acid (AA) -based exchange cooperation of microorganisms.

As basic building blocks of proteins, AAs are essential for microorganisms. However, many studies have shown that many microorganisms that survive in AA-deficient environments are AA auxotrophic (Mee et al., 2014; Embree et al., 2015; Zhu et al., 2020). This means that the microorganisms in that habitat have to obtain AAs from the rest of the members and accordingly form an important symbiosis relationship. Mee et al. (2014) constructed 14 AA auxotrophs by using *Escherichia coli*. These auxotrophs were used to construct two-, three- and fourteen-member artificial microbial floras, and the differences among the structures of these microbial floras were measured after 400 generations. It has been shown that AAs with different synthetic costs lead to different floras structures and that high synthetic-cost AAs (methionine, lysine, isoleucine, arginine, tyrosine, and phenylalanine) result in stronger cooperation. D'Souza and Kost (2016) used *E. coli* evolutionary experiments to show that after 2000 generations of evolution, AA auxotrophs appeared in all lineages, and that these auxotrophs were more adaptive. These studies demonstrated that AA auxotroph in a habitat can form a solid cooperative relationship among members and this relationship is beneficial to the community stability.

A further question is whether this AA cooperation remains robust and how the community is affected if the concentration of AAs in the environment is changed. Hoek et al. (2016) used yeast to construct two AA auxotrophs (tryptophan and leucine), and the two were able to form a solid symbiosis without AA supplementation. However, with the increase of freely available AA in the environment, this pair of strains can exhibit obligatory symbiosis, facultative symbiosis, competition, parasitism, and competitive exclusion. This confirms that the availability of AAs can change the microbial interactions and thus affect the overall stability of the community. However, this study only considered the exchange of two AAs between two members, and it is still doubtful whether this conclusion can be generalized to natural microbial communities.

In order to investigate this issue, we enriched an acetate-degrading methanogenic microbial community by operating a thermophilic methanogenic reactor fed with acetate as the sole carbon source and without exogenous AA supplementation. The microorganisms in this reactor were found to lack the ability to synthesize some AAs by metagenomic and metatranscriptomic analysis. In addition, we found that the AAs synthesis strategies of members in communities were influenced by the synthetic energy cost of AA (Akashi and Gojobori, 2002; Yao et al., 2021). The work of Akashi and Gojobori (2002) converts metabolites in the AA synthesis pathway to ATP amounts and uses the sum of ATPs as synthesis costs of AAs. As energy metabolism strategies are more important for microorganisms in energy-limited anaerobic environments, therefore, in the present study, we classified 20 AAs into four groups (low-cost AA combinations, medium-cost AA combinations, high-cost AA combinations, and all AA combinations) according to their synthetic energy cost and supplemented them separately into four methanogenic reactors fed with acetate. The differences in the structure of communities as well as the member relationships were compared. The community structure and the co-occurrence network were significantly changed in these four reactors compared to the control reactor without AA addition.

## 2 Materials and methods

### 2.1 Reactor construction and operation

Seed sludge taken from a stably operating thermophilic methanogenic chemostat fed with acetate as the sole carbon source (Zeng et al., 2024) was used to inoculate five completely mixed continuous thermophilic methanogenic reactors with a working volume of 400 ml. The reactors were fed with synthetic wastewater [total organic carbon (TOC): 8 g/L] with acetate as the sole carbon source. Detailed concentrations of inorganic salt fractions, trace elements, and vitamins in the synthetic wastewater can be found in the study by Yao et al. (2021) and Zeng et al. (2024). The reactors were operated at a dilution rate of 0.1 d^−1^ with a hydraulic retention time (HRT) of 10 days at 55°C.

Reactors A, B, C, and D were supplied with low synthetic cost-, medium synthetic cost-, high synthetic cost-AAs, and all 20 AAs, separately, after 30 d-operation. Reactor E was used as a control without AA addition. The grouping of AAs fed to the reactors and the amounts of AAs added are shown in Table 1.

**Table 1:** The strategy of AAs addition.

Amino acids	Reactor_A	Reactor_B	Reactor_C	Reactor_D	Reactor_E
Glutamate	2*	0	0	2	0
Cysteine	2	0	0	2	0
Alanine	2	0	0	2	0
Glutamine	2	0	0	2	0
Aspartate	2	0	0	2	0
Proline	2	0	0	2	0
Asparagine·H_2_O	0	2	0	2	0
Threonine	0	2	0	2	0
Glycine	0	2	0	2	0
Valine	0	2	0	2	0
Serine	0	2	0	2	0
Leucine	0	2	0	2	0
Arginine	0	2	0	2	0
Methionine	0	2	0	2	0
Lysine	0	0	2	2	0
Isoleucine	0	0	2	2	0
Tyrosine	0	0	2	2	0
Histidine	0	0	2	2	0
Phenylalanine	0	0	2	2	0
Tryptophan	0	0	2	2	0

^*^The unit of amino acid is μmol/day.

The biogas generated was collected using a drainage collection tank and the volume of biogas generated per day was counted based on the volume of water discharged. The gas components were analyzed using a gas chromatograph (GC-2014C, Shimadzu, Japan). The chromatographic column was C13X (Shimadzu, Japan, column temperature 50°C). Hydrogen was detected by a FID detector, and methane and carbon dioxide were detected by a TCD-L detector. An appropriate amount of fermentation broth was sampled and filtered through a 0.25 μm filter membrane. The filtrate was used for the measurement of the concentration of volatile fatty acids (VFAs) and TOC. VFAs were determined using a high performance liquid chromatograph (SCL-10AVP, Shimadzu, Japan) and TOC were determined using a TOC detector (TOC-VE, Shimadzu, Japan) (Zheng et al., 2019).

### 2.2 DNA extraction and 16S rRNA data analysis

During the stable operation phase of the reactors after AA supplementation, sludge samples were collected at six time points (39, 42, 48, 51, 55, and 61 days) in chronological order for DNA extraction to track changes in the microbial community structure in each reactor. DNA was extracted using the CTAB method (Yao et al., 2022). The V4-V5 variable region of 16S rRNA was amplified using the universal primers 515F (5′-GTGCCAGCMGCCGCGGTAA-3′) and 909R (5′-CCCCGYCAATTCMTTTRAGT-3′). Sequencing of these amplicons was done on the Illumina MiSeq platform and the raw data are available in NCBI Sequence Read Archive (SRA, BioProject ID: PRJNA1078993).

Analyses of microbial community species composition as well as beta diversity were done by the R package phyloseq (McMurdie and Holmes, 2013). Bray-Curtis distances among archaeal communities and among bacterial communities were calculated separately and sorted by non-metric multidimensional sorting (NMDS) to show the differences in microbial community structure among samples. The microbial co-occurrence network as well as the network characteristics analysis were conducted in iNAP (integrated Network Analysis Pipeline) (Feng et al., 2022). SparCC correlation (Friedman and Alm, 2012) was calculated using genus as the basic unit. The SparCC correlation at *r* > 0.6 and *p* < 0.05 were used for the network construction. The proportion of positive interactions for each genus in the network is defined as the value of the number of positive interactions of the genus/degree of the genus. The Greedy modularity optimization method was used to construct module separation of each network (Newman, 2004).The within-module connectivity (Z) and among-module connectivity (P) for each genus were computed and the keystones were determined accordingly (Olesen et al., 2006). The inter-group significance test in the boxplot was done by ANOVA and Tukey’s multiple tests.

## 3 Results

### 3.1 Reactor performance

**Figure 1:**
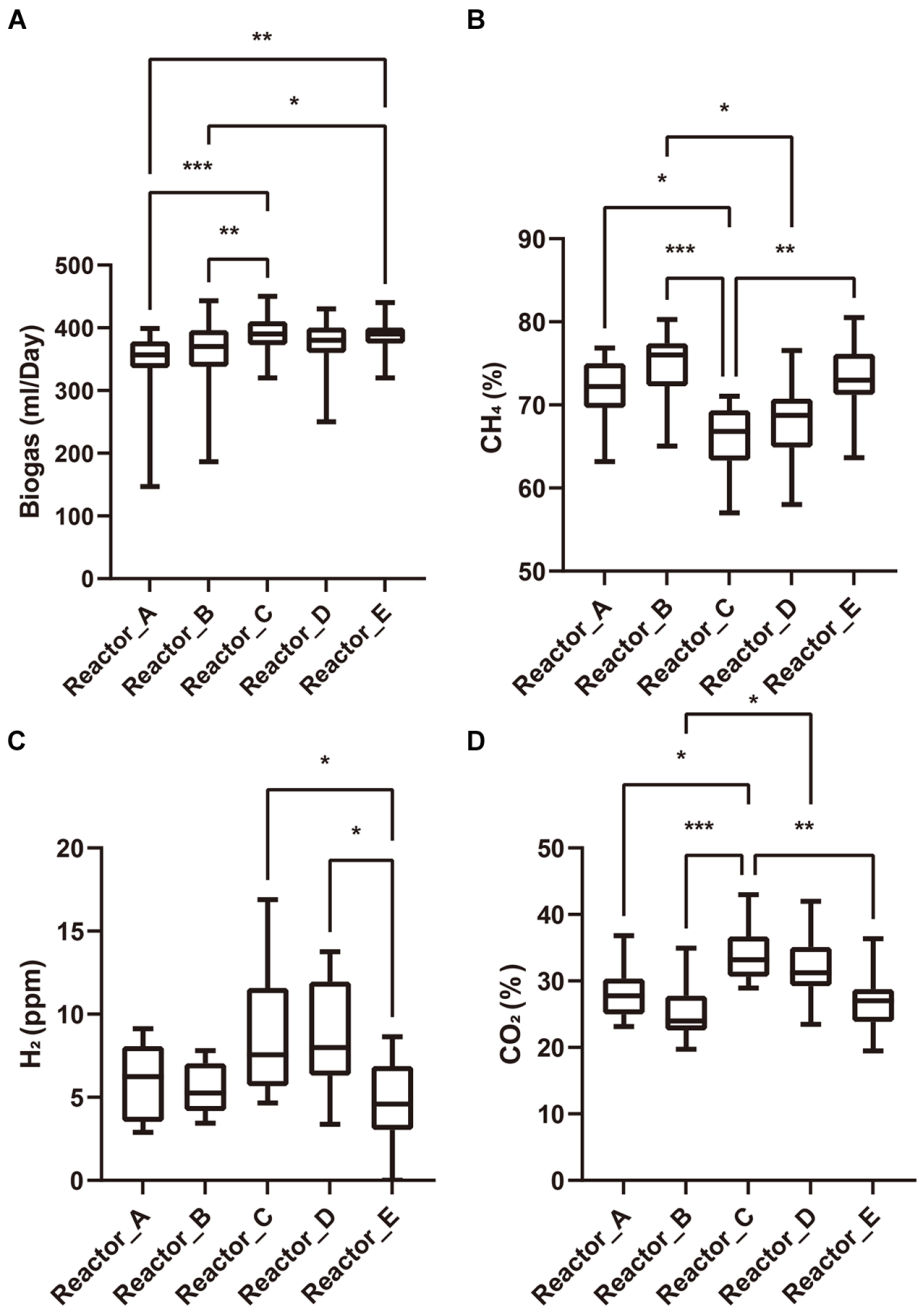
Biogas and its composition. **(A)** biogas production; **(B)** proportion of methane; **(C)** partial pressure of hydrogen; **(D)** proportion of carbon dioxide. “*”: *p* < 0.05; “**”: *p* < 0.01; “***”: *p* < 0.001. The whiskers on a box plot indicate the range from the lowest to the highest value. Bottom to top of the box indicates 25th, 50th, and 75th percentile, respectively.

AA addition to the reactors was started after three HRTs of stable operation of each reactor (no accumulation of acetate and stable biogas production). The biogas yields of reactors A, B, C, and D with AA addition decreased gradually over the next 2 days, and the accumulation of acetate occurred, whereas reactor E without AA addition did not show such a phenomenon. However, over the next six days, biogas production of reactors A, B, C and D gradually recovered and acetate ceased to accumulate. All the reactors then remained in a stable operating condition for over three HRTs. These results suggests that the AA addition produced some perturbation on the microbial communities in reactors but did not lead to the collapse of the system. We summarized the daily production of biogas and the proportions of gas components in each reactor during the stabilization phase after reactor recovery and tested the differences among reactors by Tukey’s test (Figure 1). When acetate is used as the carbon source, methane is produced through two pathways, one is that acetate can be converted to methane directedly by acetoclastic methanogens, and the other is that acetate is oxidized by syntrophic acetate-oxidizing bacteria (SAOB) to produce hydrogen and carbon dioxide which are later converted to methane by hydrogenotrophic methanogens (Schink, 1997; Yao et al., 2021). Thus, the biogas production as well as the biogas composition can reflect the activity of methanogens and bacteria in the microbial community. In terms of biogas production, reactors A and B supplemented with low-cost and medium-cost AAs, respectively, had significantly lower biogas yields than reactor C supplemented with high-cost AAs and the control reactor E. There was no significant difference among the biogas production of reactor D supplemented with all AAs and the other reactors. It is noteworthy that the proportion of methane in the biogas was significantly lower in the reactor supplemented with highly cost-AAs and all AAs than in the rest of the reactors, while the proportions of carbon dioxide and hydrogen were significantly higher than in the rest of the reactors. Differences in biogas composition among reactors supplemented with low- and medium-cost AAs and the control reactor were not significant. These differences in the biogas (yield and proportion of gas components) among the different experimental groups indicated that the AAs with different synthetic cost had different effects on the metabolism of the microbial community.

### 3.2 Microbial community structure and composition

**Figure 2:**
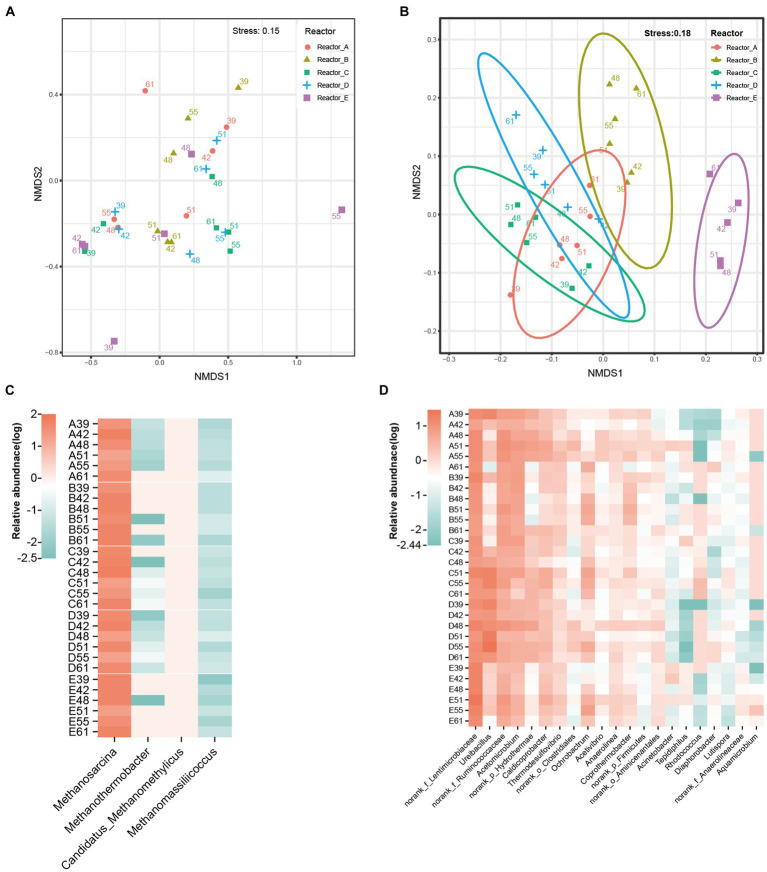
Difference of microbial communities in different reactors. **(A)** NMDS of archaeal communities, **(B)** NMDS of bacterial communities, the number of dots in the graph indicates the number of days. **(C)** archaeal genera, **(D)** major bacterial genera. norank_f_Anaerolineaceae: an unidentified genus in the family Anaerolineaceae; norank_f_Lentimicrobiaceae: an unidentified genus in the family Lentimicrobiaceae; norank_f_Ruminococcaceae: an unidentified genus in the family Ruminococcaceae; norank_o_Aminicenantales: an unidentified genus in the order Aminicenantales; norank_o_Clostridiales: an unidentified genus in the order Clostridiales; norank_p_Firmicutes: an unidentified genus in the phylum Firmicutes; norank_p_Hydrothermae: an unidentified genus in the phylum Hydrothermae.

The differences in microbial community structure in these five reactors are shown in Figures 2A,B. The effect of AA supplementation on the structure of the archaeal community was not significant, and the samples in each reactor did not show significant clustering. In addition, analysis of variance by permutation multivariate ANOVA also showed that there was no significant difference among the archaeal communities in different reactors (Supplementary Table S1). Distinctly, the bacterial communities in different reactors had different clustering patterns and were significantly different among reactors (Supplementary Table S1). According to PERMANOVA, the R2 and *p*-value among reactors A, B, C, D, and the control reactor E were 38%, 0.001; 34%, 0.001; 34%, 0.002; and 50%, 0.001, respectively. Significant differences in bacterial community structure were also observed among microbial communities supplemented with different AAs (such as reactor B and reactors A, C, and D).

Among the archaeal communities of all the reactors, the four main genera were methanogens including *Methanosarcina*, *Methanothermobacter*, *Candidatus*_Methanomethylicus, and *Methanomassiliicoccus* (Figure 2C). *Methanosarcina* was overwhelmingly dominant in the microbial community, with average relative abundances of 40.25, 40.2, 39.78, 38.45, and 41.03% in reactors A, B, C, D, and E, respectively. This was due to the fact that *Methanosarcina* is able to produce methane via both the acetate trophic pathway and the hydrogen trophic pathway and thus gains a stronger energetic advantage in acetate-fed methanogenic system. The dominant bacterial genera were mainly norank_f_Lentimicrobiaceae (an unidentified genus in the family Lentimicrobiaceae), norank_f_Ruminococcaceae (an unidentified genus in the family Ruminococcaceae), *Ureibacillus*, and *Acetomicrobium* (Figure 2D). However, the relative abundance of these bacterial genera in different reactors varied significantly. The mean relative abundance of *Ureibacillus* was 7.14, 6.04, and 14.36% in reactors A, C, and D, respectively, while it was only 0.92 and 1.46% in reactors B and E, respectively. Also with significant differences in relative abundance across reactors were *Coprothermobacter* (A, 0.70%; B, 2.52%; C, 0.90%; D, 0.60%; E, 1.06%), and *Rhodococcus* (A, 0.01%; B, 0.01%; C, 0.99%; D, 0.01%; E, 0.03%). The associated statistical tests are shown in Supplementary Table S2. Thus, the bacterial communities were more susceptible to AA supplementation than the methanogens.

### 3.3 Microbial co-occurrence network analysis

In order to investigate the changes in the interactions among members in the communities of five reactors under different AA supplementation conditions, SparCC correlation coefficients (Friedman and Alm, 2012) among the main genera of the microbial communities in the five reactors were calculated at the genus level and relationships with significance (*r* > 0.6, *p* < 0.05) were used for the construction of microbial coexistence networks (Supplementary Figure S1). The characteristics of the networks of community members in each reactor are shown in Table 2. The total nodes, total links, total positive links, and the average degree of nodes in the microbial co-occurrence networks of the four reactors with AAs supplementation were lower than those of the control reactor without AAs supplementation, especially for the reactor with all AAs supplementation followed by medium- and high-cost AAs supplementation. These may indicate that the supplementation of AAs reduced the interaction relationships among the microbial members. The AAs with different synthetic costs had different effects and AAs with low synthetic cost had the least effect.

**Table 2:** The key parameters in networks of five reactors.

Reactor	Total nodes	Total links	Total positive links	Average clustering coefficient	Average degree	Average betweenness
A	66	215	120	0.425	6.51	44.38
B	57	146	78	0.344	5.12	50.45
C	59	157	90	0.37	5.32	71.88
D	57	132	71	0.335	4.63	68.9
E	72	245	134	0.36	6.8	62.9

**Figure 3:**
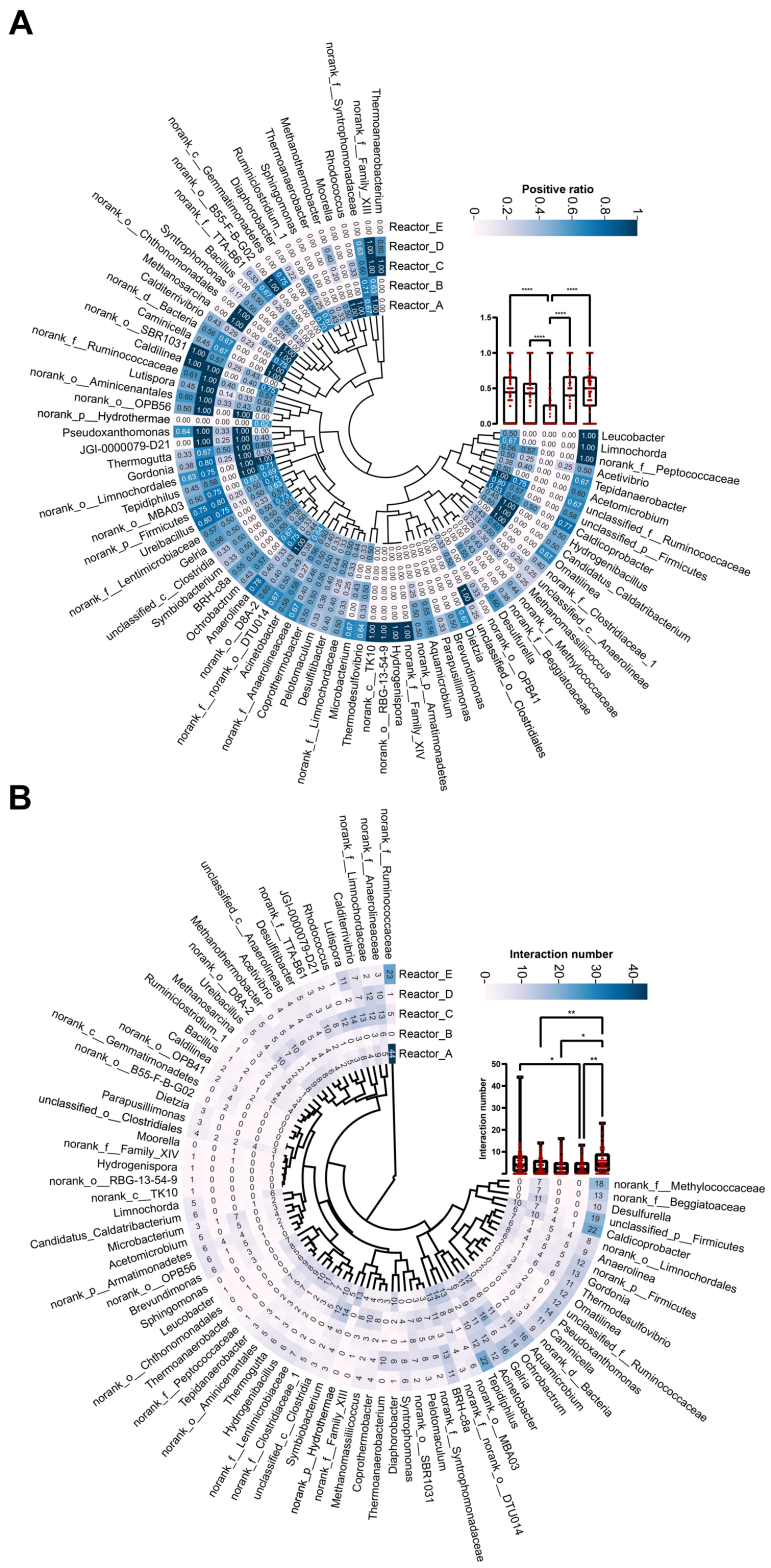
The interaction relationship among genus in each reactor. **(A)** The proportion of positive relationships of each genus. **(B)** The number of interaction relationships of each genus. “*”: *p* < 0.05; “**”: *p* < 0.01; “****”: *p* < 0.0001. The whiskers on a box plot indicate the range from the lowest to the highest value. Bottom to top of the box indicates 25th, 50th, and 75th percentile, respectively.

To further compare the variability of members interactions in different reactors, we listed the total number of interactions and the proportion of positive interactions for genera (Figure 3). The mean proportions of positive correlations for genera in reactors A, B, C, D, and E were 0.42, 0.38, 0.15, 0.4, and 0.45, respectively. It can be seen that the proportion of positive correlations among genera in reactor C supplemented with high-cost AAs was significantly lower than that in the rest of the reactors (Figure 3A). In addition, the proportion of positive correlations among genera in reactors A, B, and D was lower than that in reactor E. This may indicate that, at an overall level, AA additions reduced positive cooperation among community members, especially for those communities supplied with high-cost AAs. The average proportion of positive interactions of genus was higher in reactor D than in reactors B and C. This may be caused by a significant decrease in the degree of genus under conditions of all AAs complementation (Table 2). In terms of the number of interactions (including both positive and negative correlations) among genera in the different reactors, the mean number of interactions among genera in reactors A, B, C, D, and E were 5.1, 3.5, 3.7, 3.1, and 5.8, respectively. The number of interactions among genera was significantly higher in control reactor E than in AA-supplemented reactors especially reactor D with all AA supplementation (Figure 3B). Thus, AA supplementation reduced the interactions among genera in communities, and the effect of all AA supplementation was more significant and the low-cost AAs the least.

To further examine the differences in the interactions of the dominant genera under different AA supplementation conditions, we selected three dominant methanogen genera *Methanosarcina*, *Methanothermobacter*, and *Methanomassiliicoccus*, and three bacterial genera *Ureibacillus*, norank_f_Lentimicrobiaceae, and norank_f_Ruminococcaceae, respectively, and mapped their interactions with other genera in different reactors (Supplementary Figure S2). It was noticeable that the reciprocal profiles of each genus were significantly different in the different reactors. For example, *Methanosarcina* and *Methanothermobacter* did not interact with other genera in the control reactor E, whereas significant interactions were formed with some genera in the AA-supplemented reactors. The bacterial genus norank_f_Ruminococcaceae had significant interactions with 44 and 33 genera in reactors A and E, respectively, while no interactions were formed with any genus in reactor B, which was supplemented with medium-cost AAs. This suggests that AAs with different synthetic costs had different effects on the interactions that dominant genera formed with the remaining community members.

**Figure 4:**
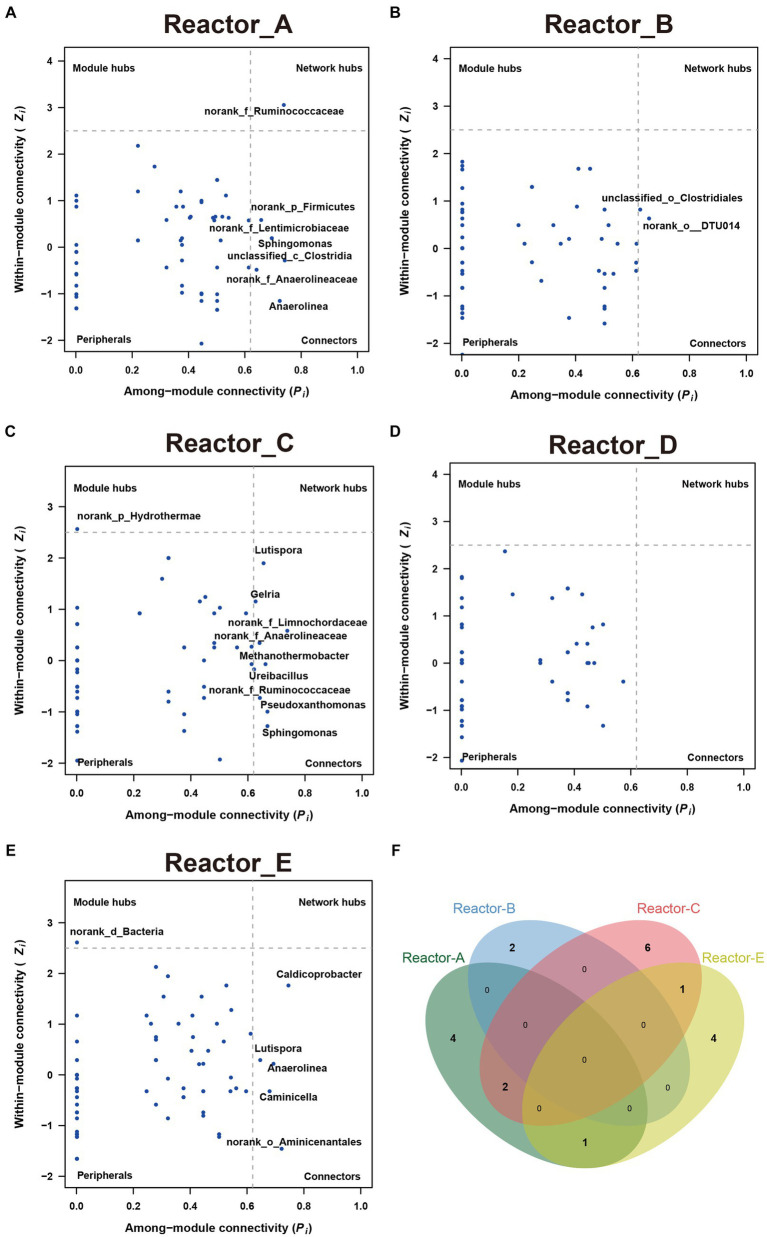
The keystones genus in communities in each reactor **(A–E)** show the key-stone genera in each reactor. **(F)** The Venn diagram of the key-stone genera (i.e., module hubs, network hubs and connectors) in reactors A, B, C and E.

To explore whether keystone genera change in the co-generation network of different reactors under different conditions of AA supplementation, the keystone genera in each system were identified by calculating the Zi (within-module connectivity) and Pi (among-module connectivity) of the different genera in each of the interaction networks (Guimerà and Nunes Amaral, 2005; Figure 4). Model hubs (high Zi, low Pi) have a high degree of connectivity only within the model they are in and little connectivity with other model hubs. Connectors (low Zi, high Pi) may play an important role in connecting other models. Network hubs (high Zi, high Pi) have many links both within the model they are in and within other models. The number of connectors in reactors A and C was relatively high for complementary low-cost and high-cost AAs. norank_f_Ruminococcaceae was a connector in reactor C, while it was a network hub in reactor A, which corresponded to its much higher number of reciprocal relationships in reactor A than in reactor C. This may indicate that low-cost AAs enhanced the ecological role of norank_f_Ruminococcaceae. In addition, the three keystones mentioned above were not present in the co-occurrence network in Reactor D, which was supplemented with all AAs. Notably, the composition of keystone genera varied considerably across reactors, with very few genera able to exist in two of the five networks (Figure 4F), suggesting that there were significant differences in the effects of AAs with different synthetic costs on the ecological status of members in a community.

## 4 Discussion

We constructed five acetate-fed methanogenic reactors. Four of them were supplemented with low-synthesis-cost AAs, medium-synthesis-cost AAs, high-synthesis-cost AAs, all 20 kinds of AAs, respectively, and one was used as a control reactor with no AA added. We compared the differences in biogas production, microbial community composition, and co-occurrence network of members during the stable operation phase of each reactor.

Differences in biogas production and composition among reactors may be due to modulation of microbial metabolic activity by different AAs rather than using AAs as an added carbon source. Because, AAs synthesis is an energy-intensive metabolic process. In an energy-limited anaerobic environment, using trace AAs (around 10^−4^ M, three orders of magnitude lower compared to the supplied carbon source acetate) as a carbon source for energy metabolism rather than protein synthesis is an unreasonable survival strategy. Compared to bacterial community, the variability in the abundance of methanogenic archaea and the interaction relationships with the rest of the members were not significant from reactor to reactor. This variability may be related to the difference in energy accessibility among methanogens and bacteria in reactors. In methanogenic reactors where acetate is the sole carbon source, the methanogen *Methanosarcina* has high acetate accessibility because of both the acetate trophic pathway and the hydrogen trophic pathways (Schink, 1997; Yao et al., 2021), leading to their absolute dominance in communities. Low energy accessibility or stress conditions promote microorganisms to interact more with each other (share more public goods, e.g., AAs, cobalamin) to cope with unfavorable conditions, which is an optimized survival strategy (Piccardi et al., 2019; Zhao et al., 2023). It is clear that the number of interaction relationships of the bacterial genera with lower energy accessibility in our reactors was significantly higher than that of *Methanosarcina* (Supplementary Figures S3a–e). In addition, *Methanosarcina* was able to synthesize most of AAs itself (Yao et al., 2021) and the high energetic accessibility makes *Methanosarcina* not highly exogenous AA dependent. Therefore, the supplementation with different cost AAs had a lower effect on the number of interactions of *Methanosarcina* than other members (Supplementary Figure S3f).

Compared to *Methanosarcina*, bacterial genera in the community (e.g., SAOB) are at a disadvantage in the competition for acetate and therefore adopt a survival strategy that reduces their ability to synthesize AAs (especially high-cost AAs). In our previous study (Yao et al., 2021), we found that SAOBs generally cannot synthesize the majority of the 20 AAs, especially those with medium and high synthetic costs. This increases the AA’s dependence of SAOBs and then reduces its metabolic activity. Under the conditions of AAs supplementation (especially with high-cost AAs), the number of interactions and the proportion of positive interactions of potential SAOBs (judging from genus names, not including other unknown potential SAOBs in the communities) with the remaining members of the community were reduced (Supplementary Figure S4). This indicates that adding AAs alleviated SAOB’s AA dependence on other members. In addition, compared to *Methanosarcina*, SAOBs, which have a lower capacity for AAs synthesis, benefited more from obtaining AAs from external AA supplementation. In turn, the metabolic activity and the competitiveness of acetate of SAOBs were therefore enhanced. Consequently, SAOBs converted more acetic acid to H_2_ and CO_2_ in the high-cost and all AAs supplemented reactors (Figure 1).

Supplementation of different cost AAs significantly reduced the level of interactions among microbial members especially when all 20 AAs were supplemented (Figure 3B). This may indicate that AA supplementation reduced the AA exchange-based cooperation among members. This phenomenon was also found in the study of Hoek et al. (2016), where the relationship among a pair of strains based on AA exchange was transformed from an obligatory mutualism to a competitive exclusion as the number of available AAs in the environment increased. In our study, the supplementation of different cost AAs all reduced the proportion of positive interactions among members, with the most significant effect being achieved by the supplementation of AAs with high synthesis cost. In our previous study, very few members in the acetate-degrading anaerobic microbial community were able to synthesize high-cost AAs (Yao et al., 2021). This phenomenon was similarly found in the study of Mee et al. (2014) and in the synthetic syntrophic community, biosynthetically costly AAs tended to promote stronger synergistic interactions. Thus, an increase in the accessibility of high-cost AAs in the environment is likely to reduce positive cooperation among members based on the exchange of high-cost AA, thus significantly reducing the number of interaction relationships among community members. This also suggests that AAs, which are more expensive to synthesize, can have a greater impact on the interactions among microbial community members.

In studying the impact of public good accessibility on microbial members’ interaction relationships and community structure, most of the previous research subjects used synthetic microbial floras formed by auxotrophs from model strains [*E. coli* (Mee et al., 2014) or *Saccharomyces cerevisiae* (Hoek et al., 2016)]. These studies enable a quantitative description of effects in terms of direct observables, such as the number of strains. Conclusions can be supported by mathematical modelling. However, these auxotrophs are genomically almost identical. This means that they overlap almost perfectly in ecological niches, resulting in intense competition among strains when the environment is altered (Hoek et al., 2016). In the present study, an acetate-degrading methanogenic microbial community enriched without AAs supply was used as the object of study. Most of members in the community were AA auxotrophs and formed AA exchange interactions with each other (Yao et al., 2021). In addition to this, there are large differences in energy acquisition among these members (Zeng et al., 2024), while avoiding overlapping ecological niches. Thus, in our results, changes in AA accessibility in the environment had a more pronounced effect on bacterial genera with low energy accessibility and did not lead to the collapse of the microbial community.

Of course, many issues are still in need of further investigation in future studies. Firstly, tracking the incorporation of stable isotope-labelled AAs in the microbial proteome may give us a deeper understanding of AAs utilization of community members. It is possible to reveal the community members who are more likely to obtain AAs from external sources and which AAs are more easily absorbed and utilized by community members. This will undoubtedly help us to better explain the effects of AAs on the structure and function of microbial communities. Secondly, considering the diversity of AAs synthetic pathways, grouping AAs by characterizing amino acid synthetic pathways (e.g., precursors) would also probably lead to interesting findings., Thirdly, the calculation of the relative abundance of microbial members has some bias. The methods such as BarBIQ (Jin et al., 2023) that can quantitatively characterize the absolute abundance of community members should be employed in the future study.

In addition, acetate-degrading methanogenic microbial community in this study is one kind of diverse natural microbial communities. Whether the conclusions in the present study can be applied to other natural microbial communities is not clear. Therefore, more microbial communities with different characteristics need to be investigated in future studies.

## 5 Conclusion

The acetate-degrading methanogenic microbial community was supplemented with different combinations of AAs with different synthesis costs. This resulted in changes to the structure of the microbial community and the interactions among microbial members. Bacterial genera which had low energy accessibility were likely affected more in interaction relationships and abundance compared to methanogens with high energy accessibility. The increase in AA accessibility reduced the number of interaction relationships within the microbial community. This effect was more significant when more kinds of AAs were supplemented. The positive correlation among community members was reduced with increased accessibility of AAs. This effect became more pronounced as the synthesis cost of AA increased.

## Data availability statement

The datasets presented in this study can be found in online repositories. The names of the repository/repositories and accession number(s) can be found at: NCBI – PRJNA1078993.

## Author contributions

JY: Writing – original draft, Writing – review & editing. QZ: Funding acquisition, Writing – review & editing. MG: Supervision, Writing – review & editing. Y-QT: Project administration, Supervision, Writing – review & editing.
