# Supplementary material for: High synthetic cost-amino acids reduce member interactions of acetate-degrading methanogenic microbial community
Source: Front Microbiol. 2024 Mar 28;15:1368215. doi: 10.3389/fmicb.2024.1368215 (PMC11007023; doi:10.3389/fmicb.2024.1368215)
Supplement: Supplementary file 1 [file Data_Sheet_1.docx]

***Supporting Information for***

**High synthetic cost-amino acids reduce member interactions of acetate-degrading methanogenic microbial community**

Jian Yao^1, 2^, Quan Zhang^3^, Min Gou^1, 2^, Yue-Qin Tang^1, 2*^

^1^College of Architecture and Environment, Sichuan University, Chengdu, Sichuan, China

^2^Sichuan Environmental Protection Key Laboratory of Organic Wastes Valorization, Chengdu, Sichuan, China

^3^Dalian Petrochemical Research Institute of China Petroleum and Chemical Corporation, Dalian, Liaoning, China

^*^Correspondence: Yue-Qin Tang

Tel. (fax): +86 28 85990936

E-mail: tangyq@scu.edu.cn


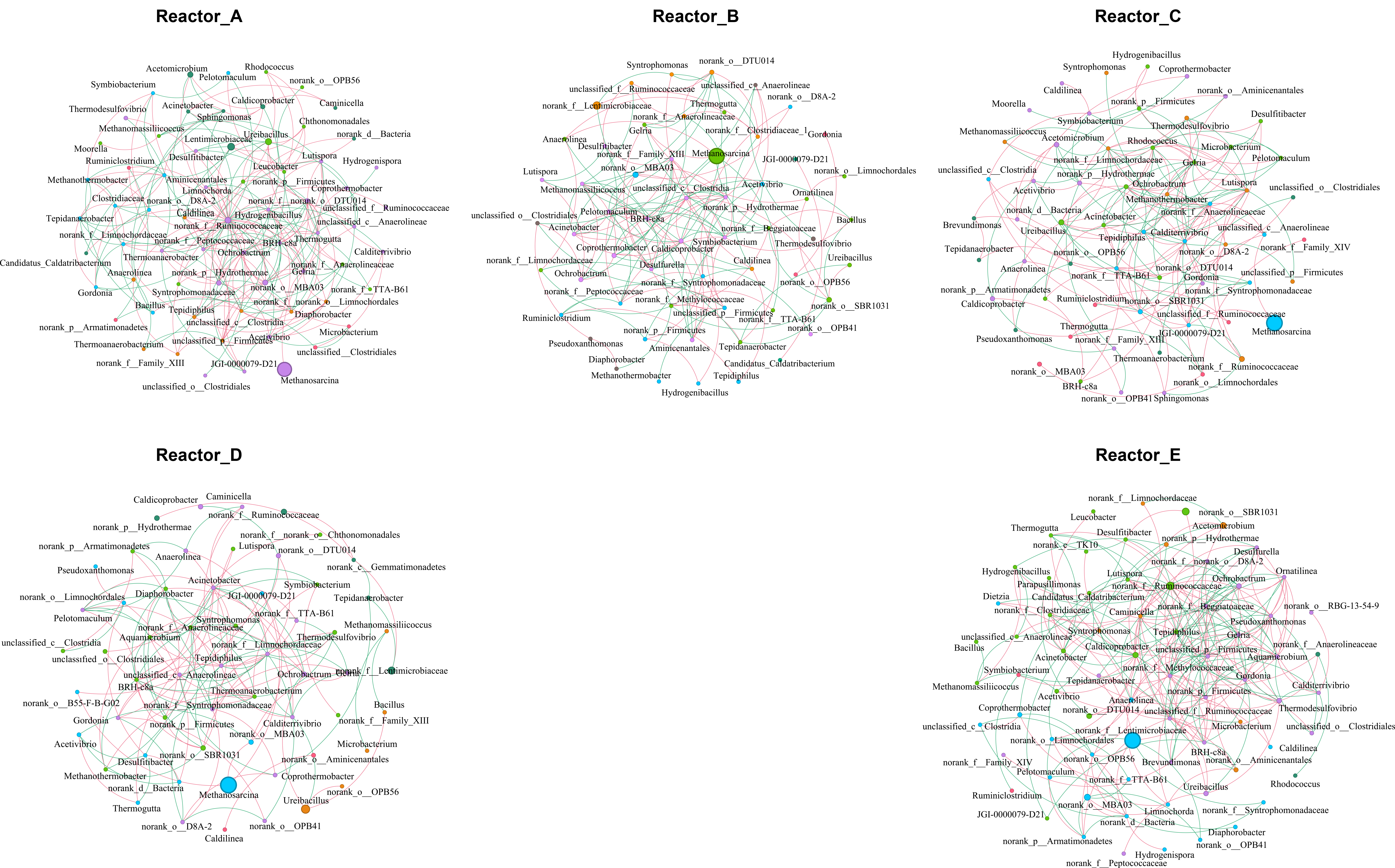


Fig S1 The co-occurrence network of each reactor

The color of a node represents the module in which the node is located. The size of the nodes indicates the relative abundance of the genu. The green edge indicates a negative interaction relationship; the red edge indicates a positive interaction relationship.


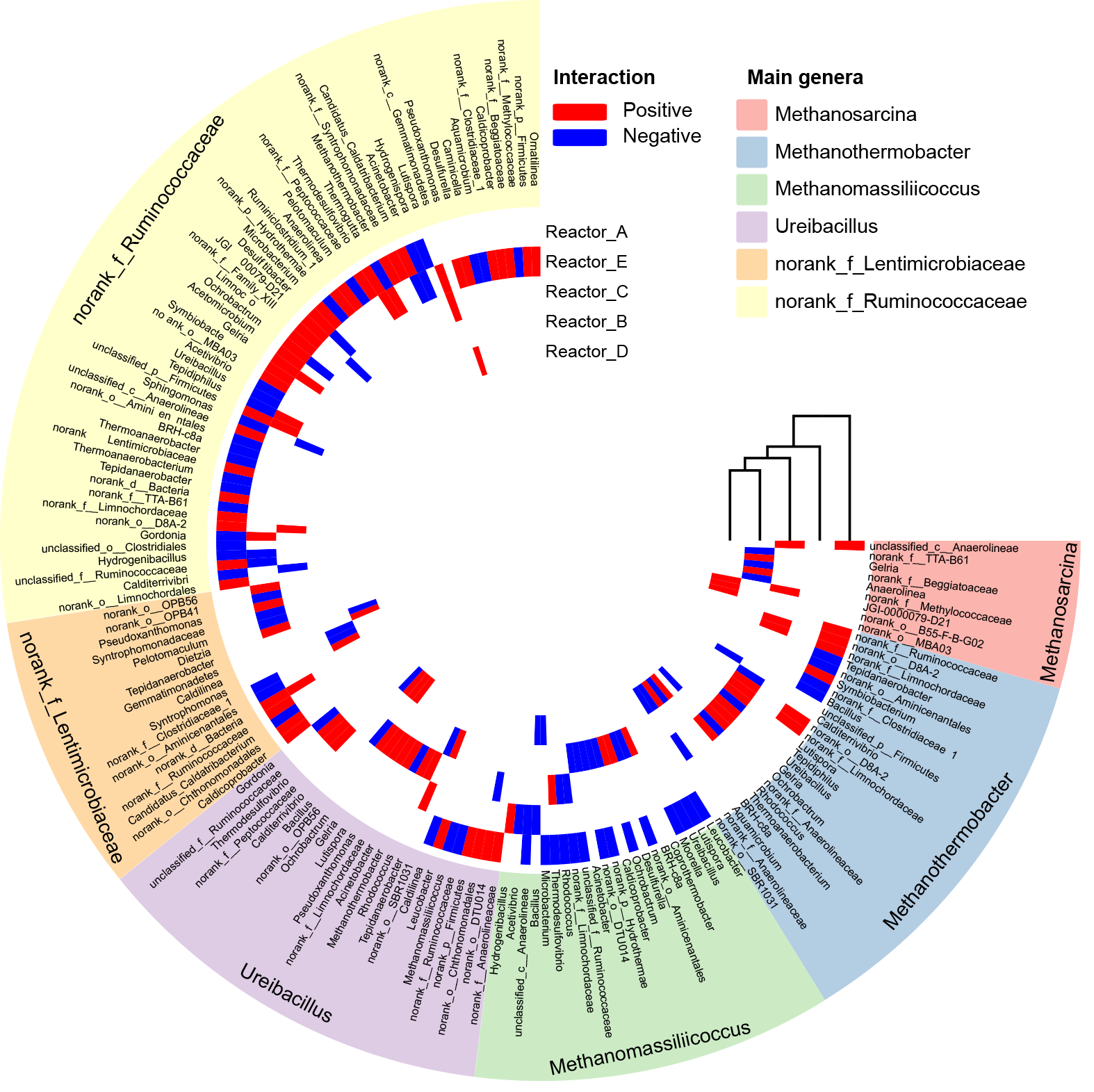


Figure S2 The positive and negative interaction relationships of major genera

f__Lentimicrobiaceae: an unidentified genus in the family Lentimicrobiaceae (phylum Bacteroidetes); norank_f__Ruminococcaceae: an unidentified genus in the family Ruminococcaceae (phylum Firmicutes). The genus in the colored blocks are the genus that interact with the target genera. For example, the genus in the blue color block is the genus that interacts with *Methanothermobacter*.


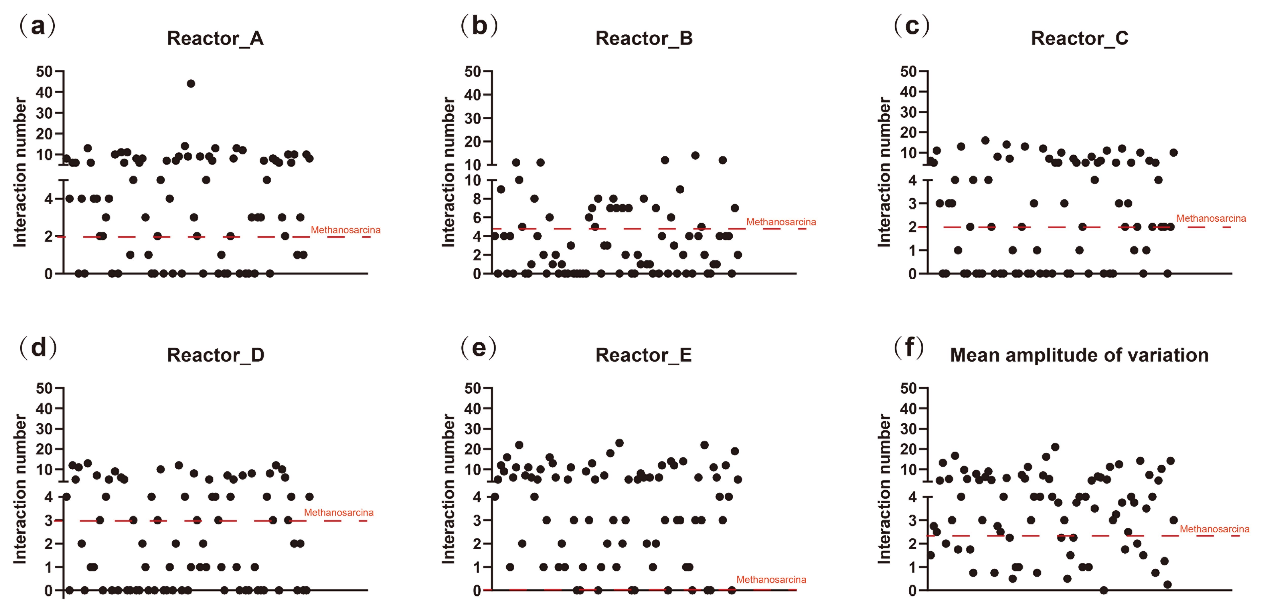


Figure S3 The number of interactions of each genus and the mean amplitude of variation relative to the Reactor_E.


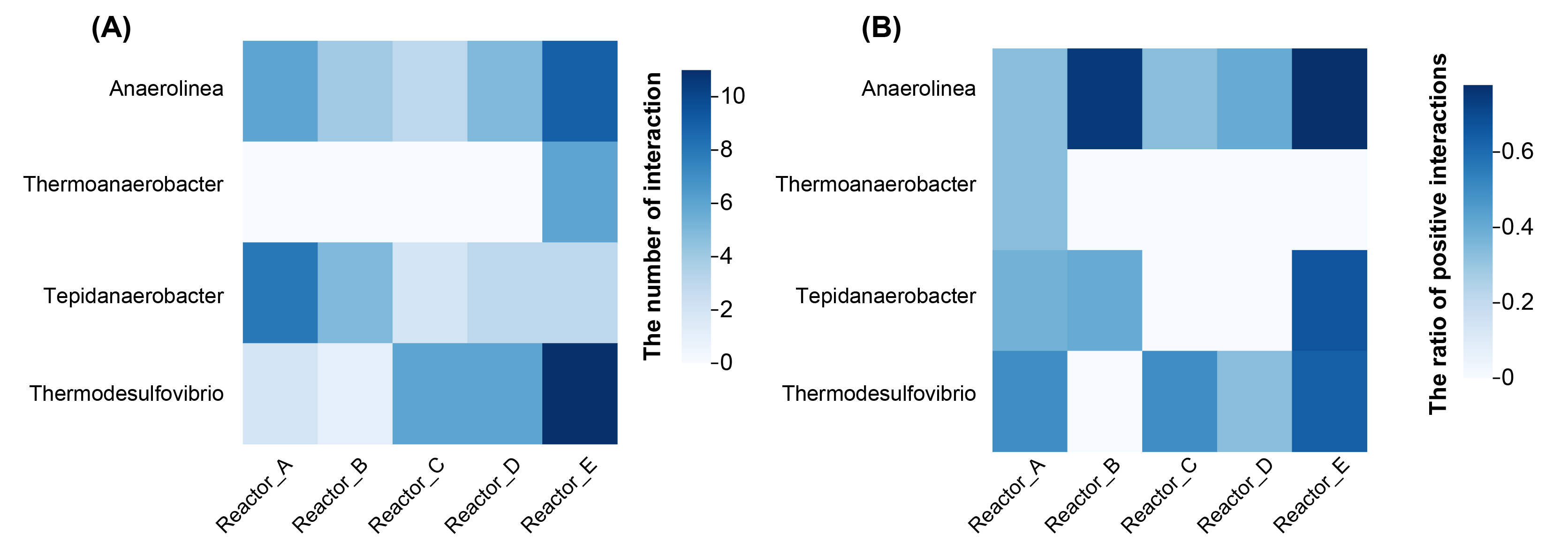


Figure S4 The interaction relationships of syntrophic acetate oxidating bacterial genus. (A) The number of interactions among SAOBs and other community members; (B) The ratio of positive interactions among SAOBs and other community members.

Table S1 PERMANOVA of bray-curtis distance between different reactors

| Archaeal community | | | Bacterial community | |
| --- | --- | --- | --- | --- |
| Pairwise | R2 | Pr(>F) | R2 | Pr(>F) |
| A / B | 0.008 | 0.874 | 0.300 | 0.001 |
| A / C | 0.032 | 0.502 | 0.091 | 0.170 |
| A / D | 0.004 | 0.890 | 0.178 | 0.027 |
| A / E | 0.003 | 0.925 | 0.384 | 0.001 |
| B / C | 0.016 | 0.763 | 0.257 | 0.002 |
| B / D | 0.021 | 0.676 | 0.467 | 0.001 |
| B / E | 0.007 | 0.918 | 0.348 | 0.001 |
| C / D | 0.052 | 0.376 | 0.150 | 0.044 |
| C / E | 0.019 | 0.626 | 0.341 | 0.002 |
| D / E | 0.010 | 0.763 | 0.501 | 0.001 |

Table S2 The ANVOA of different genus in different reactors

|  | Ureibacillus | o__Clostridiales | p__Hydrothermae | Coprothermobacter | Rhodococcus |
| --- | --- | --- | --- | --- | --- |
| A vs. B | 0.3119 | 0.6968 | <0.0001 | <0.0001 | >0.9999 |
| A vs. C | 0.9969 | 0.2701 | 0.2018 | 0.9786 | <0.0001 |
| A vs. D | 0.179 | 0.8887 | >0.9999 | 0.9962 | >0.9999 |
| A vs. E | 0.4015 | 0.2145 | <0.0001 | 0.8396 | >0.9999 |
| B vs. C | 0.507 | 0.9474 | 0.0379 | <0.0001 | <0.0001 |
| B vs. D | 0.0011 | 0.1946 | <0.0001 | <0.0001 | >0.9999 |
| B vs. E | 0.9998 | 0.9082 | 0.9928 | <0.0001 | >0.9999 |
| C vs. D | 0.088 | 0.0389 | 0.1944 | 0.8821 | <0.0001 |
| C vs. E | 0.6119 | >0.9999 | 0.0124 | 0.9905 | 0.0001 |
| D vs. E | 0.0019 | 0.028 | <0.0001 | 0.6345 | >0.9999 |

o_Clostridiales: an unidentified genus in the order Clostridiales (phylum Firmicutes); p__Hydrothermae: an unidentified genus in the phylum Hydrothermae (phylum Hydrothermae)
